# Trauma care and development assistance: opportunities to reduce the burden of injury and strengthen health systems

**DOI:** 10.2471/BLT.18.213074

**Published:** 2019-04-01

**Authors:** Barclay Stewart, Sara Hollis, Stas Salerno Amato, Eileen Bulger, Charles Mock, Teri Reynolds

**Affiliations:** a Department of Surgery, University of Washington, 1959 NE Pacific Street, Suite BB-487, PO Box 356410, Seattle, WA 98195-6410, United States of America (USA).; b Department for the Management of Noncommunicable Diseases, Disability, Violence and Injury Prevention, World Health Organization, Geneva, Switzerland.; c Department of Surgery, University of Vermont Medical Center, Burlington, USA.

Injury is one of the most neglected health crises of our time, yet in 2106 it accounted for 32% more than the global number of fatalities that result from malaria, tuberculosis and human immunodeficiency virus/acquired immunodeficiency syndrome (HIV/AIDS) combined.^1^ The effects of early mortality and prolonged disability due to injury also mean that injury places staggering economic burdens on countries.^2^ For instance, the annual cost of road traffic injuries alone, which constitute less than a third of injuries globally, is approximately 1.0% of the gross national product in low-income countries, 1.5% in middle-income countries, and 2.0% in high-income countries.^3^

Notwithstanding the magnitude of the problem, well-established and relevant solutions exist, including organized trauma care. Organized trauma care is a coordinated effort to deliver the full spectrum of care to injured patients, from the time of the injury through community reintegration.^4^ The organization of pre-hospital care, acute care (that is, hospital-based care, including emergency, critical and surgical care) and rehabilitation services is typically administered by a public agency. The responsibilities of such agencies are to provide leadership, governance and appropriate finances, support prevention initiatives, coordinate service delivery, establish minimum standards of care, designate trauma centres, enable quality improvement programming and ensure system evaluation.

Organized trauma care, particularly when combined with multisectoral injury control initiatives, saves lives, limits disability, promotes productivity and lessens individual, community and national economic burdens. However, despite the human costs of trauma and the existence of evidenced-based and affordable interventions, national and global health agendas have failed to give priority to injury control and trauma care.^2^^,^^3^^,^^5^

## Development assistance

To implement resolutions addressing care of the injured, the United Nations (UN) General Assembly resolution A/RES/58.289, the World Health Assembly resolutions WHA57.10, WHA60.22 and WHA68.15, and given the evidence demonstrating the cost–effectiveness of trauma care,^2^^,^^4^^,^^6^ more must be done to finance and organize such care. In upper middle- and high-income countries, regional or national trauma systems are being established or maturing, which has resulted in marked reductions in preventable death and disability.^4^^,^^6^ In many low- and middle-income countries, most trauma care is provided by ad hoc and under-resourced systems, owing, in part, to insufficient national resources or diversion of funds to other sectoral priorities.^4^ To mitigate resource constraints in these countries, which harbour more than 90% of the global injury burden,^4^ significant development assistance for trauma care, domestic resource mobilization and technical support infrastructure may prove useful. Although there is no consensus concerning the effect of development assistance for health, evidence suggests that it has significant and positive impacts on health outcomes in recipient countries.^7^ The sustainable development goal (SDG) 10, reducing inequality within and among countries, directs the global development community to encourage official development assistance and financial flows, including foreign direct investment, to countries where the need is greatest.

Much is known about the landscape of development assistance for health in general, in particular for high-profile global health conditions such as HIV, tuberculosis, malaria, and maternal and child health; however, the funding landscape for trauma care is less defined. Development assistance for health should be systematically reviewed to promote transparency and hold donors and recipient countries accountable to population and recipient countries’ needs. However, examination of development assistance financing flows highlights several gaps about development assistance for trauma care.^8^ First, such assistance is not aligned with national or global disease burdens. Second, development assistance for trauma care is critically underfunded despite numerous evidence-based, highly cost–effective interventions that strengthen health systems broadly. Third, this assistance may be limited by a lack of injury-related technical expertise within donor and channel entities, that is, intermediaries between donors and recipients.

Ideally, development assistance for health would be dispersed in proportion to burden of disease and recipient national health system needs, and associated with an accountability framework.^9^ However, development assistance for trauma care has been estimated to represent less than 1.0% of all health assistance, while HIV/AIDS, tuberculosis, and malaria received 36.0% of such assistance in 2017.^8^ That is, trauma care received 0.04 United States dollars (US $) per disability-adjusted life year (DALY) incurred, while the corresponding amount for HIV/AIDS was US $ 4.05, tuberculosis US $ 25.09, malaria US $ 9.62 and maternal and child health US $ 45.75. Over the last three decades, this development assistance has resulted in remarkable gains related to disease-specific prevention and health system strengthening efforts, leading to reductions in the burdens of these conditions.^10^ On the other hand, in low- and middle-income countries, increases in injuries related to the epidemiological transition, motorization and development, ageing, violence, and failure to prioritize injury control and trauma care have created a health crisis.

## Domestic resource mobilization

Several concerns about development assistance for health have been raised. There is evidence that recipient countries have reallocated some of their domestic resources away from health to other sectors following inflows of such assistance. Other case studies have suggested that development assistance for health imposes substantial costs on recipient countries, partly because each donor has different application, monitoring and reporting requirements. There have also been concerns about temporal stability of health assistance given global financial crises, and how these might impact recipient countries’ health systems and their population health when such assistance is reduced. To mitigate some of these concerns, the development community has called for increases in domestic resource mobilization to match or contribute to common donor and recipient health priorities.

Domestic financing has increased in many countries with high burdens of injury, and in many cases, is no longer inconsequential compared to development assistance for health. Much might be gained from mobilizing domestic resources for trauma care, which may also have positive effects beyond improving care for the injured.^11^ Such resources would allow governments to deliver an essential public good, trauma care, thereby strengthening national ownership of this health focus. Additionally, domestic resource mobilization is a facet of the broader good governance for health agenda that is required to catalyse greater progress towards universal health coverage (UHC) and health-related sustainable development goals.

In low- and middle-income countries, the deficits in trauma care relate to information, coordination, capacity and research. These gaps could be addressed with better planning and organization and only modest investments of domestic resources and/or development assistance for health.^4^ To achieve SDG 17.1, that is, “to strengthen domestic resource mobilization, including through international support to developing countries, to improve domestic capacity for tax and other revenue collection,” low- and middle-income countries could consider action. For example, additional taxes on alcohol, firearms, vehicle registration earmarked for injury control and trauma care organization, value-added tax that goes towards UHC, or tax incentives for trauma system capital investments. Similarly, development assistance for health disbursements and public-private partnerships could be designed to incentivize mobilization of domestic resources through matching programmes.

## Technical expertise

Evidence exists around injury control and organization of trauma to guide strategic disbursement of scarce funding.^4^^,^^6^ Since injury has not been prioritized, existing donors and channels have limited technical expertise and infrastructure to identify potentially successful and/or cost–effective interventions and programming. Injury control and trauma care technical expertise within government agencies, national and international professional societies, the World Health Organization (WHO), health institutes and academia could be leveraged to maximize the effect of health assistance for trauma care. Such action would result in the reduction of the injury burden and the strategic development of comprehensive emergency and trauma care systems.

## Trauma care, UHC and SDGs 

All UN Member States have agreed to achieve UHC by 2030. Providing care for the injured without risk of catastrophic health expenditure represents a unique opportunity to reduce the burden of injury and strengthen health systems more broadly. Improvements in trauma care can contribute to several SDGs (Table 1).

**Table 1:** Opportunities to address the sustainable development goals by supporting injury control initiatives and organized trauma care

SDG	Specific goal	Role of injury control and organized trauma care in achieving the specific goal
3.4	By 2030, reduce by one third premature mortality from noncommunicable diseases through prevention and treatment and promote mental health and well-being	Recognize injury as a noncommunicable disease; support injury prevention and surveillance systems; organize trauma care to minimize preventable death and disability; provide comprehensive rehabilitation programmes
3.5	Strengthen the prevention and treatment of substance abuse, including narcotic drug abuse and harmful use of alcohol	Prevent injuries that would require opiate pain medications and put patients at risk of addiction; employ emergency unit harm reduction interventions; incorporate substance abuse programming into rehabilitation for alcohol- and/or drug-related injuries
3.6	By 2020, halve the number of global deaths and injuries from road traffic accidents	Support multisectoral road injury prevention programmes; develop and organize post-crash trauma care
3.8	achieve universal health coverage, including financial risk protection, and access to quality essential health-care services	Include emergency conditions and injuries into risk-pooling schemes to eliminate catastrophic health expenditure; include essential trauma care and essential surgery as components of national health plans; create pre-hospital care systems; build trauma care capacity at first-level hospitals to improve timely access to care
3.D	Strengthen the capacity of all countries, in particular developing countries, for early warning, risk reduction and management of national and global health risks	Support emergency preparedness activities; plan for surge capacity in organized trauma systems
11.5	By 2030, significantly reduce the number of deaths and people affected by disasters	Build resilient emergency and trauma care systems; plan for surge capacity in organized trauma systems

SDG: sustainable development goal.

Beyond the direct benefits of organizing trauma care (for example reducing preventable death and disability after injury), the platforms that provide care for the injured, such as emergency care, acute and critical care, surgical care and rehabilitation, also improve outcomes for a range of other high-priority conditions that benefit from systematic surveillance, prompt diagnosis, timely multidisciplinary treatment and rehabilitation, including sepsis, pregnancy complications, acute infections, outbreaks and exacerbations of noncommunicable diseases. The potential for these integrated care platforms to address a multitude of SDGs is further incentive to invest and implement organized care for the injured.

## Way forward

Several opportunities to increase funding for injury control and trauma care, advocate for inclusion of injury in UHC and promote proportionally appropriate disbursement of development assistance for health for trauma care are available. National governments and the global health community should advocate for development assistance for health commensurate with disease burdens and current health system gaps. For example national governments could mobilize domestic resources through changes in prioritization and mechanisms related to injury. Development assistance for health should support low- and middle-income countries in strengthening essential trauma care, as outlined by WHO and the World Bank, in efforts to achieve UHC and progress towards the SDGs. Finally, donors and channels of such assistance should seek or develop injury control and trauma care technical expertise to guide disbursements towards high-impact, cost–effective interventions.
